# Roles of Resolvins in Chronic Inflammatory Response

**DOI:** 10.3390/ijms232314883

**Published:** 2022-11-28

**Authors:** Chang Liu, Dancai Fan, Qian Lei, Aiping Lu, Xiaojuan He

**Affiliations:** 1 Law Sau Fai Institute for Advancing Translational Medicine in Bone and Joint Diseases, School of Chinese Medicine, Hong Kong Baptist University, Hong Kong, China; 2 National TCM Key Laboratory of Serum Pharmacochemistry, Laboratory of Metabolomics, Department of Pharmaceutical Analysis, Heilongjiang University of Chinese Medicine, Harbin 150040, China; 3 Institute of Basic Research in Clinical Medicine, China Academy of Chinese Medical Sciences, Beijing 100700, China; 4 Shanghai Guanghua Hospital of Integrated Traditional Chinese and Western Medicine, Institute of Arthritis Research, Shanghai Academy of Chinese Medical Sciences, Shanghai 200052, China; 5 Guangdong-Hong Kong-Macau Joint Lab on Chinese Medicine and Immune Disease Research, Guangzhou 510120, China

**Keywords:** resolvins, chronic inflammation response, characteristics, mechanism, clinical translation

## Abstract

An inflammatory response is beneficial to the organism, while an excessive uncontrolled inflammatory response can lead to the nonspecific killing of tissue cells. Therefore, promoting the resolution of inflammation is an important mechanism for protecting an organism suffering from chronic inflammatory diseases. Resolvins are a series of endogenous lipid mediums and have the functions of inhibiting a leukocyte infiltration, increasing macrophagocyte phagocytosis, regulating cytokines, and alleviating inflammatory pain. By promoting the inflammation resolution, resolvins play an irreplaceable role throughout the pathological process of some joint inflammation, neuroinflammation, vascular inflammation, and tissue inflammation. Although a large number of experiments have been conducted to study different subtypes of resolvins in different directions, the differences in the action targets between the different subtypes are rarely compared. Hence, this paper reviews the generation of resolvins, the characteristics of resolvins, and the actions of resolvins under a chronic inflammatory response and clinical translation of resolvins for the treatment of chronic inflammatory diseases.

## 1. Introduction

The inflammatory response is the defense response of the body’s normal tissue to a damage factor such as a pathogen invasion [1]. Under normal circumstances, the inflammatory response is beneficial for the organism, but a chronic inflammatory response can result when a disorder of an inflammation resolution causes an excessive uncontrolled inflammatory response [2,3]. A chronic inflammatory response is dominated by hyperplasia, usually with lymphocyte and a plasma cell infiltration as the main pathological manifestation [4]. A chronic inflammatory disease is a chronic inflammatory response which is caused by an abnormal immune response and environmental factors including rheumatoid arthritis, atherosclerosis, diabetes, nervous system diseases, etc., which seriously threaten human health [5]. Therefore, an excessive inflammatory response must be resolved to prevent the onset of chronic inflammatory diseases and the restoration of the homeostasis of organisms [6,7,8]. The inflammatory resolution phase is characterized by the ceasing of a neutrophil infiltration, the recession of proinflammatory cytokines, and the elimination of fragments and foreign matters in the inflammatory response by efferocytosis [9,10]. Resolvins, acting as an "agonist", are metabolites of omega-3 fatty acids and together with martins, protectins, and lipoxins, they are collectively referred to as "special pro-decomposition mediators" (SPMs). The generation of resolvins begins with the peak period of inflammation and resolvins also promote an inflammation resolution better than its lipid precursors at concentrations as low as one which is nanomolar [11,12].

Resolvins such as the E series from eicosapentaenoic acid (EPA), D series from docosahexaenoic acid (DHA), and T series from docosapentaenoic acid (DPA) are synthesized from essential omega-3 fatty acids by corresponding enzymes such as epoxidase and lipoxidase [13,14]. Omega-3 fatty acid has been proved to be effective in treating COVID-19 [15], cardiovascular diseases [16], cognitive impairment [17], rheumatoid arthritis [18], and other complex diseases through an immune regulation. The actions of resolvins in the inflammatory response mainly include reducing the PMN inflammatory infiltration [19,20,21,22], decreasing inflammatory factors [23,24,25,26,27,28], improving inflammatory pain and preventing a central sensitization [29]. The basic characteristics and biological effects of common resolvins have been demonstrated. Since earlier studies were conducted on individual subtypes of resolvins, using a different experimental design, a comparison of the results is possible. Therefore, this paper reviews the possible effect of resolvins on promoting an inflammation resolution under chronic inflammatory response state and explores their clinical use to resolve inflammation.

## 2. Production and Characteristics of Resolvins

Resolvins are the products of omega-3 polyunsaturated fatty acids DHA, EPA, and DPA metabolic catalyzed by lipoxygenases (LOXs), cytochrome P450s (CYP450s), and cyclooxygenases (COXs) [30,31]. The D-series resolvins have been synthesized from RvD1 to RvD6, and the E series have been synthesized from RvE1 to RvE4 and 18S-RvE1. The T series of resolvins have also been discovered and synthesized from RvT1 to RvT4 [32].

**Figure 1:**
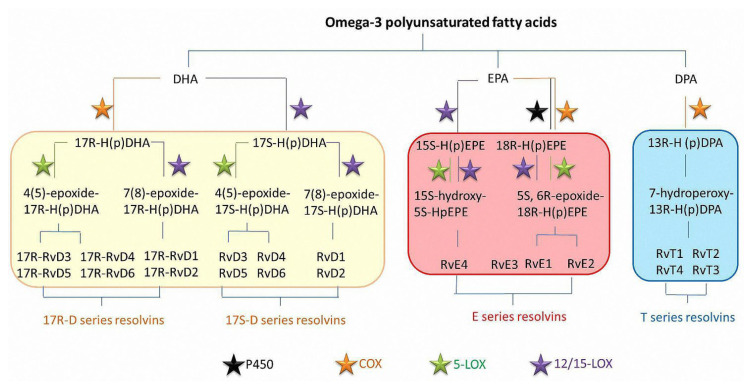
The generation of D series resolvins, E series resolvins, and T series resolvins.

As shown in Figure 1, there are two routes for the synthesis of D series resolvins. One route is that exogenous DHA stored in cell membranes is catalyzed by 15-lipoxygenase (15-LOX) into 17S-H (p) DHA [33]. On the one hand, 17S-H (p) DHA is further converted into 7(8)-epoxide intermediates by 15-LOX and becomes RvD1 and RvD2 by the related enzymes [34,35]. RvD1 and RvD2 were first detected in human cord blood using LC-MS/MS [36]. On the other hand, 4-(5)-epoxide intermediates are generated through 5-LOX, and RvD3-RvD6 are generated under the action of enzymes [37,38]. RvD1 to RvD6 belong to the 17S-D series of resolvins. RvD3 and RvD4 have been detected in human bone marrow cells based on metabololipidomics [39,40]. Through the method of untargeted lipidomics, RvD5 in a synovial fluid gathered from rheumatoid arthritis patients was also detected [41]. Another synthetic route is the conversion of DHA to 17R-H(p)DHA by acetylated-COX-2 (acCOX-2), which is then catalyzed to the 17R-D series of resolvins by LOXs. Although the 17R-D series of resolvins are initially named aspirin-triggered resolvins D (AT-RvDs) as their biosynthesis is initiated by aspirin, recent research shows that the COX-2 can be acetylated by other agents in vivo, so these resolvins are named 17R resolvins D according to the IUPAC nomenclature of the stereocenters [42,43]. These resolvins have the same effects of promoting an inflammation resolution as the 17S-D series resolvins in vivo [44].

The biosynthesis of E-series resolvins normally occurs under the action of endothelial cells and leukocytes, and EPA is converted to 18R-H(p)EPE by ASACOX-II. With the help of 5-LOX, 18R-H(p)EPE enters the cell directly and is converted into 5S-peroxy-18R-hydroxy-EPE [45]. In the end, the 5(S),6-epoxy intermediate is converted to RvE1 [46]. At the same time, the complete stereochemical structure of RvE1 was determined to be 5S,12R,18R-trihydroxy-6Z,8E,10E,14Z,16E-EPA [47]. However, RvE2 is produced after the hydrolysis of the 5(S),6-epoxy intermediate, and RvE3 is generated after 18R-H(p)EPE is catalyzed by 12/15-LOX [48]. By 15-LOX, EPA is converted to 15S-H(p)EPE and further converted to 15S-hydroxy-5S-HpEPE, the precursor of RvE4 with the help of 15-LOX or 5-LOX [49]. In addition, another synthetic pathway for E-series resolvins is the conversion of EPA to 18R-H(p)EPE and 17,18-epoxyeicosatetraenoic acid(17,18-EEP) by cytochrome P450 oxidase [50].

The T series of resolvins are derived from DPA via endothelial cyclooxygenase-2 (COX-2). The first precursor during the production of T series resolvins is 13(R)-hydroperoxy-docosapentaenoic acid and it is converted to 13(R)-hydroxy-docosapentaenoic acid after a reduction [51]. When converted to 7-hydroperoxy-13(R)-hydroxy-docosapentaenoic acid, RvT4 is obtained from the reduction in the hydroperoxy group after the transcellular trafficking to the neutrophils [52]. The enzymatic lipoxygenase reaction occurs during the transition to RvT1, and an allylic epoxide intermediate is formed in the process of the conversion to RvT2 and RvT3 [53]. T series resolvins are either generated in healthy subjects or are converted from DPA after statin therapy [4,54]. The complete stereochemical structure of resolvins is shown in Table 1.

## 3. Resolvins Receptors

RvD1, 17R-RvD1, RvD3, 17R-RvD3, RvD5, RvE1, and 18S-RvE1 can combine with the G protein-coupled receptor (GPR32) expressed in monocyte-macrophages, lymphocytes, endothelial cells, and vascular smooth muscle cells, while GPR18/DRV2 is a novel discovered receptor of RvD2 expressed in macrophages, neutrophils, and monocytes [55,56]. In addition, RvD1, 17R-RvD1, and RvD3 can also combine with the G protein-coupled formyl peptide receptor 2 (ALX/FPR2) to produce anti-inflammatory effects [57,58,59]. Leukotriene B4 receptor 1 (BLT1) expressed in lymphocytes and leukocytes and ChemR23 expressed in NK cells, macrophages, epithelial cells, and dendritic cells have been proven to be the receptors of RvE1, 18S-RvE1, and RvE2 through target screening [60,61]. The actions of RvD4 in the phagocytosis of human leukocytes have been suggested to be related to Gs protein and the responding G protein-coupled receptors [39]. The resolvin receptors are cell specific and organ specific and have been shown to be affected by disease and homeostatic states. However, the corresponding receptors of the characterized RvD6, RvE3, and T series of resolvins have not yet been identified, and relevant studies are still needed to explore these receptors [62].

## 4. Effects of Resolvins on Target Cells in Chronic Inflammatory Response

**Figure 2:**
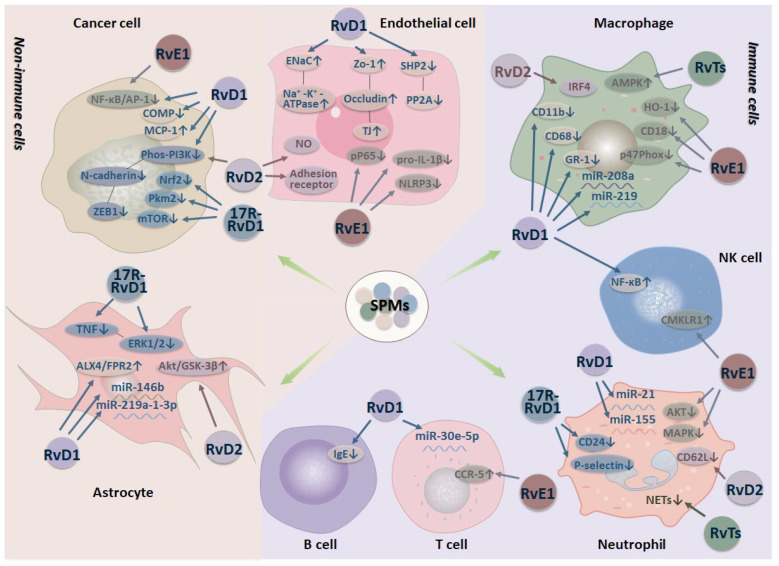
Different actions of resolvins on several target cells.

The cellular targets of resolvins depend on the unique receptor repertoire of the different cells. Different resolvin subtypes also have common target cells, while the mechanisms of their actions on the same cell have obvious differences, as shown in Figure 2.

### 4.1. Effects on Neutrophils

Neutrophils constitute the cell’s first line of immune defense. Activated neutrophils may release neutrophil extracellular traps (NETs) during a distinct form of cell death, which promote inflammation. RvE1 regulates CD18 in neutrophils, downregulates the phosphorylation of AKT and MAPK, and inhibits a superoxide production and migration between epithelial and endothelial cells [63,64]. RvD1 stops neutrophil chemotaxis, inhibits a neutrophil migration, and downregulates the actin aggregation and the expression of adhesion molecules via miR-21 and miR-155 [65]. 17R-RvD1 decreases the expression of p-selection and its ligand CD24 in neutrophils, while RvD2 decreases CD62L shedding in human neutrophils [66]. Bacterial and viral infections trigger an uncontrolled inflammation upon contact with excess NETs and clearance obstacles of NETs. The newly found T series resolvins dose- and time-dependently reduce NETs in human neutrophils and restricted neutrophil infiltration and NETs in a murine *Staphylococcus aureus* infection [67].

### 4.2. Effects on Macrophages

Macrophages produce a large number of inflammatory cytokines during autoinflammatory diseases that cause a cascade of release of inflammatory mediators and are therefore regarded as inflammatory triggers [68]. Macrophages are critical for restoring homeostasis, and their function contributes to the pathological development of fibrosis, atherosclerosis, cancer, and other chronic diseases in the event of a persistent injury or failure to resolve inflammation [69]. RvE1, RvD1, RvD2, RvD3, and RvD5 can all act on macrophages, but their effects are exerted in different ways. RvE1 reduces the expression of the HO-1 and NADPH oxidase subunit p47Phox, while RvD1 induces the transition from M1 macrophages to M2 macrophages [70,71]. RvD2 regulates the transcription factor IRF4, while RvD3 and RvD5 enhance efferocytosis and phagocytosis of M2 macrophages [34,55]. Monocytes are the precursors of macrophages, and the functions of RvE1 on monocytes are to decrease the shedding of L-selectin and the expression of integrin CD18, while RvD1 decreases CD11b, CD68, and GR-1 [72]. RvD1 and RvD2 regulate a macrophage polarization through multiple targets, while the activation of the PKA pathway is necessary [73,74]. RvD1 can regulate miR-208a and miR-219 in macrophages to target regulating IL-10 and 5-lipoxygenase [58,75]. RvT2 in the T series stimulates the clearance of NETs by macrophages through increasing intracellular cyclic adenosine phosphate and phospho-AMP-activated protein kinase (AMPK) in both human and mouse cells [67].

### 4.3. Effects on Lymphocytes

Lymphocytes are the smallest leukocytes and include T cells, B cells, and natural killer (NK) cells. A lymphocyte infiltration is a key step in the tissue damage caused by a chronic inflammatory response in which lymphocytes migrate abnormally into nonlymphoid tissues [76]. B cells are the target cells of RvD1, which reduces their production of IgE, while RvD2 protects against an alveolar bone loss by decreasing CD4^+^ T cells [77,78]. RvD5 has been assessed to significantly inhibit the Th17 cells’ differentiation and proliferation, and RvD1 controls downstream miR-30e-5p to increase Treg and reduce the differentiation of Th17 to repair the imbalance in the Treg/Th17 ratio [79,80]. RvE1 not only inhibits the proliferation of Th17 but it also affects Th17s secretion of IL-17 and increases the expression of CCR-5 [81]. In addition, RvE1 facilitates the cell migration of NK to remove eosinophilic granulocytes and apoptotic PMNs by CMKLR1, and RvD1 also reduces the secretion of TNF-α and IL-6 in acinous cells by upregulating NF-κB [82,83,84].

### 4.4. Effects on Astrocytes

Astrocytes are the most widely distributed cells in the brain and dynamically regulate a neural and synaptic plasticity, the blood-brain barrier (BBB) homeostasis, and the transmission of signals [85] that are closely related to inflammation and Alzheimer’s disease [86]. Astrocytes promote and maintain a microglial activation, leading to a chronic inflammatory response, in addition to regulating the activity of oligodendrocytes and cells of the adaptive immune system and controlling an infiltration of the central nervous system [87,88]. RvD1 can activate the ALX4/FPR2 receptor in astrocytes and induce higher levels of mitochondrial phagocytosis to eliminate damaged mitochondria [89]. Another study showed that RvD1 impaired a neuronal cell death through miR-146b and miR-219a-1–3p to enhance functional recovery [26]. 17R-RvD1 has effects on the neuronal dysfunction by impairing and downregulating the neuronal plasticity [90]. It can not only decrease the release of TNF in LPS and IFN-γ-stimulated astrocytes, but also reduce the TNF-induced activation of ERK1/2 [91]. Zhang et al. showed that GPR18 was expressed in astrocytes, and the activation of RvD2-GPR18 mediated an anti-nociceptive effect by regulating the Akt/GSK-3β signal pathway in a radicular pain rat model [92].

### 4.5. Effects on Endothelial Cells

Endothelial cells, which are found at the inner wall of the blood vessels, are the target of RvD1, RvD2, and RvE1. The junction of interendothelial adhesion molecules forms the endothelial cell barrier that regulates the vascular permeability and leukocyte extravasation. An increased or prolonged leukocyte recruitment leads to the increased expression of interendothelial adhesion molecules during the chronic inflammatory response [93]. RvD2 regulates the production of NO and the expression of adhesion receptors in endothelial cells [94]. RvD1 activates the ENaC and Na^+^-K^+^-ATP axes by regulating the ALX/cAMP/PI3K signaling pathways and effectively blocks the interaction between endothelial cells and monocytes as well as the inactivation of SHP2 and PP2A [95]. In addition, RvD1 upregulates the expression of ZO-1, occludin, and tight junction proteins to protect against the impairment of the endothelial barrier function through the IκBα pathway [96]. RvE1 has been demonstrated to attenuate an endothelial senescence by reducing not only the expression of the pro-IL-1β proteins pP65 and NLRP3 but also the generation of the NLRP3 inflammasome complexes [97].

### 4.6. Effects on Cancer Cells

The steps of chronic inflammation-induced cancer syndrome include a cell transformation, promotion, survival, proliferation, invasion, angiogenesis, and metastasis [98]. Resolvins specifically inhibit the cancer progression stimulated by cell fragments through a reprogramming of macrophages to enhance the clearance of fragments, and counter regulating cytokines or chemokines released by human macrophages [74,99]. In addition, resolvins can stimulate the antineoplastic activity of neutrophils [100]. Resolvins also inhibit the differentiation of cancer-associated fibroblasts in a hepatocellular carcinoma and pancreatic cancer [101,102]. Besides affecting the function of immune cells in a tumor microenvironment, resolvins can also directly act on tumor cells. When acting on A549 lung cancer cells, RvD1 and RvD2 inhibit the epithelial–mesenchymal transition (EMT) by reducing the phosphorylation of PI3K to downregulate the expression of N-cadherin and ZEB1 and modulate the macrophage polarization to inhibit cancer growth [74,103,104]. RvD1 inhibits the secretion of a cartilage oligomeric matrix protein (COMP) by targeting FPR1/ROS/ROXM1 signaling, thereby impeding the cancer stem-like properties and EMT of hepatocellular carcinoma cells [101]. Moreover, increasing the expression of monocyte chemoattractant protein-1 (MCP-1), stimulating PMNs reprogramming, and promoting a protective PMN-dependent recruitment are potential ways that RvD1 inhibits tumor growth [100]. RvE1 and RvD1 reduce the activity of NF-κB/AP-1 to prevent the transition from a liver injury and hepatitis to liver cancer [105]. The anticancer actions of resolvins, mainly including RvD1, RvD2, and RvE1, are achieved with the receptor-dependent promotion of a tumor debris clearance and the inhibition of tumor cell growth mediated by cells in the tumor stroma [99]. However, 17R-RvD1 inhibits EMT by reducing the expression of mTOR, Pkm2, and Nrf2 to downregulate E-cadherin and vimentin [20].

## 5. Mechanism and Clinical Translation of Resolvins for Chronic Inflammatory Diseases Treatment

Due to the fact that the receptors of resolvins are located on different target cells, tissues, and organs, different resolvins have different effects on different diseases. Increasing evidence and research have been provided to clarify the relationship between resolvins and the chronic inflammatory response as well as chronic inflammatory diseases. Therefore, resolvins have been gradually applied in clinical practice based on their mechanism.

### 5.1. Effects on Pain

Research on heat pain sensitivity and osteoarthritis pain in 250 healthy volunteers and 62 volunteers affected with knee osteoarthritis was performed to measure endogenous 17-HDHA, RvD1-3, RvD5, and RvE1 by LC-MS. The study concluded that the level of 17-HDHA was closely related to increasing heat pain thresholds, and its effect on the sensitivity of the heat pain and osteoarthritis pain was based on the level of DHA [106]. A mouse model of postoperative pain induced by a tibial bone fracture (fPOP) was used to show that RvD1 and RvD5 could reduce mechanical allodynia and cold allodynia and that RvD1 inhibited mechanical hyperalgesia by regulating CGPR [107]. Further research indicated that RvD1 increased a cell-selective polarization by regulating the NK-κB nuclear translocation and M2-type polarization in microglial cells, thereby reducing the neuropathic pain [108]. RvD2 alleviated hyperalgesia in cystitis rats by activating GPR18 and inhibiting TRPV1, and RvD5 exhibited sex differences in the modulation of pain and inhibited neuropathic pain only in male mice [109,110]. The ERK signaling pathway is a common target regulated by RvD1 and RvE1, while RvD1 significantly alleviates neuropathic pain by inhibiting the Nod-like receptor protein 3 (NLRP3) inflammasome in the ERK signaling pathway, and RvE1 alters the mechanical allodynia by inhibiting the ERK phosphorylation in spinal dorsal horn neurons [111,112]. RvD1 and RvE1 have analgesic effects on bone cancer pain, and RvD1 can additionally promote the upregulation of endocannabinoids through CB2 receptors, so RvD1 has a stronger analgesic effect on bone cancer than RvE1 [113].

### 5.2. Effects on Atherosclerosis

Fat-fed Ldlr−/− mice were administered RvD1 to test whether SPMs, especially RvD1, were related to facilitating efferocytosis in atherosclerosis and suppressing plaque necrosis by a targeted a mass spectrometry platform. Finally, the research confirmed that RvD1 had the ability to promote a plaque stability by accelerating lesional efferocytosis and decreasing lesion oxidative stress and necrosis [114]. To examine whether RvE1 could reduce atherosclerosis, a mouse model of ApoE*3 Leiden mice fed a hypercholesterolaemic diet was used, and the RvE1 effects of protecting against atherosclerosis and decreasing a lesional inflammation had been demonstrated to involve regulating the expression of immune responses and inflammatory genes in arteries without changing the serum amyloid A and plasma cholesterol [115]. The proliferation and migration of vascular smooth muscle cells (VSMCs) is a significant feature of the atherosclerotic lesions. Research shows that RvE1 can block the migration of the human great saphenous vein mesangial cells stimulated by a platelet-derived growth factor and reduce the phosphorylation of the platelet-derived growth factor receptor-β to control the formation of atherosclerosis [116]. RvE1 inhibits the contractions of both the human pulmonary artery (HPA) and rat thoracic aorta (RTA), while RvD1 and RvD2 only control the contractions of the RTA. These results indicate that resolvins provide useful novel therapeutics and have the characteristic of increasing the activity of thromboxane contractile for pulmonary arterial hypertension [11]. Viola et al. have demonstrated that the delivery of RvD2 and Maresin 1 induces macrophages to transform into the repair phenotype and stimulate the collagen synthesis of smooth muscle cells [117]. Elajami et al. tested the endogenous SPMs profiles of six patients suffering from stable coronary artery disease (CAD) through targeted metabololipidomics, and the results showed that the absence of RvE1, RvD1, RvD2, RvD3, and RvD5 appeared in CAD patients. After a treatment with Lovaza (one capsule contains 840 mg of EPA and DHA), 17R-RvD3 and RvD6 were increased in patients with CAD, and the result also showed that SPMs could promote a macrophage phagocytosis in blood clots [118,119]. In addition, RvD1 was able to reduce M1-type macrophages in patients with ApoEε3/ε3 but increased M1-type macrophages in patients with ApoEε3/ε4 [17]. Resolvins, as natural receptor agonists mainly associated with the stability of plaques and a lack of proatherogenic side effects, have a great potential as a new therapy for CAD [120]. Patients with Chagas disease always develop chronic myocarditis when the infection is untreated. 17R-RvD1 modulates a local and systemic inflammation in mouse cardiac tissue chronically infected with parasites, reduces cellular infiltration, and reduces cardiac hypertrophy and fibrosis in the early chronic phase of the disease, providing a strategy for improving drug therapy [121].

### 5.3. Effects on Diabetes

In conjunction with the interactions among adipocytes, macrophages, and other immune cells infiltrating the adipose tissue, adipose tissue produces an inflammatory response, which in turn contributes to the development of type 2 diabetes mellitus (T2DM) by leading to an insulin resistance [122]. Research was performed with twenty-four T2DM patients randomized to a three-period crossover study including drinking red wine for 4 weeks or the equivalent volumes of water or dealcoholized red wine to measure the effects of red wine on plasma SPMs of T2DM patients and the difference between patients and healthy volunteers. Red wine did not appear to differentially affect any SPMs, and the SPM levels in T2DM patients were higher than those in healthy volunteers. This research proves that increasing the SPM level is a homeostatic response to counter ongoing inflammation [123]. In addition, by researching dorsal root ganglion (DRG) neurons in a T2DM mouse model fed with a high-fat diet for 8 weeks and administered daily injections of RvD1, the results showed that RvD1 could promote the growth of DRG neurites and indicated that neuropathy, such as the slow nerve conduction caused by T2DM, could be reversed by RvD1 [124]. The results showed that a supplementation with polyunsaturated fatty acids could be an effective therapy for diabetic neuropathy. The most recent research showed that the MAPK-NF-κB signaling pathway was the target of RvD1 for fungal growth and the inflammatory response against Aspergillus fumigatus keratitis in diabetes [125]. RvD5 prevents the elevation of IL-1β in the hippocampus and prefrontal cortex of type 1 diabetes mellitus rats, thereby intervening in behaviors related to depression and anxiety. RvE3 plays a role in glucose homeostasis by activating PI3K and Akt phosphorylation to enhance the adipocyte uptake of glucose [126].

### 5.4. Effects on Anti-Depression and Wound Healing

Different resolvin subtypes also play the same role through different mechanisms. In terms of anti-depression, RvD1 plays an anti-depression role through the transmission of glutamic acid or PI3K/AKT signaling, while RvD2 can also act on MEK/ERK [127]. The activation of mTORC1 signaling is the key anti-depressive role of RvD1, RvD2, RvE1, RvE2, and RvE3 [25,128,129,130,131,132]. In addition, RvD2 promotes the healing of periapical bone lesions and the regeneration of pulp-like tissue by enhancing the expression of dentin matrix acidic phosphoprotein 1 (DMP1) and the phosphorylation of STAT3 [133]. To promote wound healing, the effect of RvE1 is obviously better than that of RvD2, while the effect of RvD1 is weaker than that of RvD2 [134]. Resolvin D6-isomer (RvD6si) contributes to the functional recovery and wound healing of corneal tissue by restoring an innervation after injury [135].

### 5.5. Effects on Rheumatoid Arthritis

Rheumatoid arthritis (RA) is characterized by chronic inflammation and progressive joint destruction. The levels of RvD1 and RvE1 in patients with RA had been measured and were significantly lower than those in healthy individuals and patients in remission of RA [136]. RvD1 restrains the expression of TRAP, cathepsin K, TNF-α, IL-1 β, IFN-γ, and PGE2 to inhibit the differentiation and activation of osteoclasts [137]. The mechanism of RvD1 against RA had been further elucidated in a collagen-induced arthritis (CIA) mouse model; that is, RvD1 alleviated the progression of RA by inhibiting CTGF via the upregulation of miRNA-146a-5p [138]. Research has indicated that the level of RvD3 was reduced in delayed-resolving arthritis mice and RA patients and RvD3 could reduce joint leukocytes [40]. Furthermore, an SKG arthritic mouse model was used to explore the actions of RvD5, and the results showed that RvD5 weakened the osteoclast differentiation to interfere with the genesis of osteoclasts involved in the pathogenesis of RA [79]. Another research indicated that GPR101 was the top candidate receptor for RvD5 to display antiarthritic action by regulating neutrophil and macrophage responses [139]. RvE1 inhibits bone resorption and osteoclastogenesis through suppressing the expression of the transcription factors nuclear factor of activated T cells c1 (NFATc1) and c-fos [140].

### 5.6. Application of Resolvins in the Treatment of Pre-Diseases

A clinical study aimed to evaluate the SPM levels in offspring supplied with fatty acids during pregnancy and at 12 years of age. This was the first time that RvE1, RvE2, RvE3, RvD1, 17R-RvD1, and RvD2 had been identified in human cord blood. A supplementation with n-3 fatty acids in pregnancy can significantly increase SPM precursors and DHA-derived 17-HDHA at birth but not at 12 years [36]. The application of the accumulative score relies on the gene expression related to the generation, metabolism, and signaling of D series resolvins to comparatively analyze the relationship between the resolution of inflammation and tumorigenesis. As a result, a higher RvD score distinguishes patients with an improved resolution of inflammation who can benefit from an anticancer treatment [141]. Regardless of the method of directly supplementing exogenous resolvins or increasing endogenous resolvins, resolvins have certain effects on the resolution of inflammation and the maintenance of homeostasis in vivo. Numerous animal and clinical experiments have provided the basis for the development of new anti-inflammatory drugs and the treatment of chronic diseases caused by inflammation.

## 6. Further Perspectives

Resolvins are a series of endogenous lipid mediators with a positive effect on inflammation resolution, and their origin and chemical structure have been confirmed for many years. Studies of receptors have reported a few receptors for resolvins; however, the receptors of the T-series resolvins RvD6 and RvE3 are not clear, so a further study of resolvin receptors can provide new evidence and directions for us to resolve inflammation. Many human and animal experiments have also substantiated that resolvins can reduce the joint pain and stiffness of RA, reduce vessel inflammation in coronary heart disease, promote plaque stability, and improve the metabolic parameters and complications of diabetes. The mechanism of action of the resolvins in the process of an inflammation resolution is complex, and the existing evidence is only the tip of the iceberg. A large number of studies still need to be confirmed and explained.

In inflammatory diseases, there are naturally expressed resolvins in many organs. However, as its biosynthetic precursor omega-3 polyunsaturated fatty acid cannot be synthesized by the human body itself, the resolvins obtained from the body are very limited. As resolvins are endogenous lipid mediators and are sensitive to light, heat, and oxidation, synthesis costs are very high in vitro and production supplies are limited, so it is difficult to perform a large-scale production and new drug industry chain formation. According to systems’ biology theory and methods, resolvins can be regarded as the breakthrough point in the search for related correlative proteins and signaling pathways to elucidate the mechanism of anti-inflammatory prescriptions and drugs. The cross-analysis of multiple layers and multi-omics may be a way to provide a basis for new anti-inflammatory drugs research to discover novel drugs with fewer side effects and significant therapeutic effects on chronic inflammatory diseases as soon as possible.

## 7. Conclusions

With the development of detection techniques and a deeper understanding of the chronic inflammatory response, the study of resolvins will no longer be limited to simple individual subtypes of resolvins. Hence, this paper reviews not only the production process and characteristics of resolvins but also possible mechanisms by the subtypes of resolvins in a chronic inflammatory response and explores their clinical use to resolve inflammation.

## Figures and Tables

**Table 1:** The complete stereochemical structure and related abbreviation of resolvins.

Name	Structure	Abbreviation
Resolvin D1	7S, 8R, 17S-trihydroxy-docosa-4Z, 9E, 11E, 13Z, 15E, 19Z-hexanoic acid	RvD1
Resolvin D2	7S, 16R, 17S-trihydroxy-docosa-4Z, 8E, 10Z, 12E, 14E, 19Z-hexanoic acid	RvD2
Resolvin D3	4S, 11R, 17S-trihydroxy-docosa-5Z, 7E, 9E, 13Z, 15E, 19Z-hexanoic acid	RvD3
Resolvin D4	4S, 5R, 17S-trihydroxy-docosa-6E, 8E,10Z, 13Z, 15E, 19Z-hexanoic acid	RvD4
Resolvin D5	7S, 17S-dihydroxy-docosa-4Z, 8E, 10Z, 13Z, 15E, 19Z-hexanoic acid	RvD5
Resolvin D6	4S, 17S-trihydroxy-docosa-5E, 7Z, 10Z, 13Z, 15E, 19Z-hexanoic acid	RvD6
17R-Resolvin D1	7S, 8R, 17R-trihydroxy-docosa-4Z, 9E, 11E, 13Z, 15E, 19Z-hexanoic acid	17R-RvD1
17R-Resolvin D2	7S, 16R, 17R-trihydroxy-docosa-4Z, 8E, 10Z, 12E, 14E, 19Z-hexanoic acid	17R-RvD2
17R-Resolvin D3	4S, 11R, 17R-trihydroxy-docosa-5Z, 7E, 9E, 13Z, 15E, 19Z-hexanoic acid	17R-RvD3
17R-Resolvin D4	4S, 5R, 17R-trihydroxy-docosa-6E, 8E,10Z, 13Z, 15E, 19Z-hexanoic acid	17R-RvD4
17R-Resolvin D5	7S, 17R-dihydroxy-docosa-4Z, 8E, 10Z, 13Z, 15E, 19Z-hexanoic acid	17R-RvD5
17R-Resolvin D6	4S, 17R-trihydroxy-docosa-5E, 7Z, 10Z, 13Z, 15E, 19Z-hexanoic acid	17R-RvD6
Resolvin E1	5S, 12R, 18R-trihydroxy-eicosa-6Z, 8E, 10E, 14Z, 16E-pentaenoic acid	RvE1
18S-Resolvin E1	5S, 12R, 18S-trihydroxy-eicosa-6Z, 8E, 10E, 14Z, 16E-pentaenoic acid	18S-RvE1
Resolvin E2	5S, 18R-dihydroxy-eicosa-6E, 8Z, 11Z, 14Z, 16E-pentaenoic acid	RvE2
Resolvin E3	17R, 18R-dihydroxy-eicosa-5Z, 8Z, 11Z, 13E,15E-pentaenoic acid	RvE3
Resolvin E4	5S, 15S-dihydroxy-eicosa-6E, 8Z, 11Z, 13E, 17Z-pentaenoic acid	RvE4
Resolvin T1	7S,13R,20S-trihydroxy-8E,10Z,14E,16Z,18E-docosapentaenoicacid	RvT1
Resolvin T2	7S,12R,13S-trihydroxy-8Z,10E,14E,16Z,19Z-docosapentaenoic acid	RvT2
Resolvin T3	7S,8R,13S-trihydroxy-9E,11E,14E,16Z,19Z-docosapentaenoic acid	RvT3
Resolvin T4	7S,13R-dihydroxy-8E,10Z,14E,16Z,19Z-docosapentaenoic acid	RvT4
